# Clinical presentation and outcome of riboflavin transporter deficiency: mini review after five years of experience

**DOI:** 10.1007/s10545-016-9924-2

**Published:** 2016-03-14

**Authors:** Bregje Jaeger, Annet M. Bosch

**Affiliations:** Department of Pediatric Neurology, Emma Children’s Hospital, Academic Medical Center, Meibergdreef 9, 1105 AZ Amsterdam, The Netherlands; Department of Pediatrics, Emma Children’s Hospital, Academic Medical Center, Meibergdreef 9, 1105 AZ Amsterdam, The Netherlands

## Abstract

**Introduction:**

Riboflavin (vitamin B2) is absorbed in the small intestine by the human riboflavin transporters RFVT1 and RFVT3. A third riboflavin transporter (RFVT2) is expressed in the brain. In 2010 it was demonstrated that mutations in the riboflavin transporter genes SLC52A2 (coding for RFVT2) and SLC52A3 (coding for RFVT3) cause a neurodegenerative disorder formerly known as Brown-Vialetto-Van Laere (BVVL) syndrome, now renamed to riboflavin transporter deficiency. Five years after the diagnosis of the first patient we performed a review of the literature to study the presentation, treatment and outcome of patients with a molecularly confirmed diagnosis of a riboflavin transporter deficiency.

**Method:**

A search was performed in Medline, Pubmed using the search terms ‘Brown-Vialetto-Van Laere syndrome’ and ‘riboflavin transporter’ and articles were screened for case reports of patients with a molecular diagnosis of a riboflavin transporter deficiency.

**Results:**

Reports on a total of 70 patients with a molecular diagnosis of a RFVT2 or RTVT3 deficiency were retrieved. The riboflavin transporter deficiencies present with weakness, cranial nerve deficits including hearing loss, sensory symptoms including sensory ataxia, feeding difficulties and respiratory difficulties which are caused by a sensorimotor axonal neuropathy and cranial neuropathy. Biochemical abnormalities may be absent and the diagnosis can only be made or rejected by molecular analysis of all genes. Treatment with oral supplementation of riboflavin is lifesaving. Therefore, if a riboflavin transporter deficiency is suspected, treatment must be started immediately without first awaiting the results of molecular diagnostics.

## Introduction

### Riboflavin

Riboflavin (7,8-dimethyl-10-ribityl-isoalloxazine, vitamin B2) is a water soluble vitamin which is the precursor of the active coenzymes flavin mononucleotide (FMN) and flavin adenine dinucleotide (FAD), important cofactors for carbohydrate, amino acid and lipid metabolism (Lienhart et al. 2013). Humans depend on the dietary intake of riboflavin from milk, meats, fatty fish and green vegetables and the recommended daily allowance of Riboflavin varies from 0.4 mg (infants) to 1.6 mg (lactating women) (Powers 2003). Excess riboflavin, FAD and FMN are excreted in the urine. Besides the major supply from the diet unknown amounts of riboflavin are generated by bacteria and absorbed in the large intestine (Said 2015).

### Riboflavin transporters

**Fig. 1:**
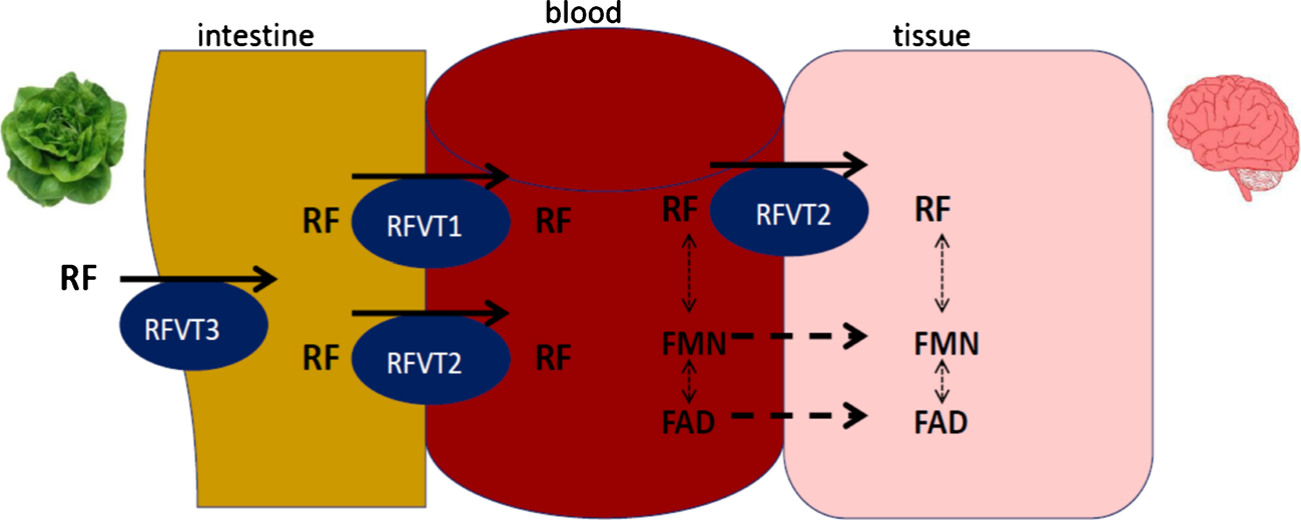
riboflavin transport (adapted from Yonazewa et al. 2008)

Riboflavin is a well-known chaperone effective in the treatment of inborn errors of metabolism such as MADD and mitochondrial disorders (Cornelius et al. 2012; Carrozzo et al. 2014). Only since 2010 there is insight into inborn errors of riboflavin transport (Green et al. 2010; Bosch et al. 2011; Bosch et al. 2012; Foley et al. 2014). Presently three riboflavin transporters have been characterized: RFVT1 mostly expressed in small intestine, RFVT 2 mostly expressed in the brain and RFVT 3 mostly expressed in the small intestine (Fig. 1, adapted from Yonazawa 2013) (Yonezawa et al. 2008; Yamamoto et al. 2009; Yao et al. 2010).

### Riboflavin transporter deficiency

In 2010 it was demonstrated that mutations in the riboflavin transporter genes SLC52A2 (coding for RFVT2) and SLC52A3 (alias C20orf54, coding for RFVT3) are the cause of the neurodegenerative disorder formerly known as Brown-Vialetto-Van Laere (BVVL) or Fazio Londe (FL) syndrome (Green et al. 2010; Bosch et al. 2011). Remarkably, except for one report of riboflavin deficiency in an infant of a mother with one mutation in the RFVT1 gene, no patients with RFVT1 deficiency have been reported in the literature.

Inheritance of RFVT2 and RFVT3 deficiency is autosomal recessive. A literature review of 75 patients with BVVL or FL syndrome demonstrated that patients may present at all ages, with a variable but mostly rapid progression and a fatal outcome if untreated, with the most severe course in the youngest age groups. Our index patient presented at the age of 6 months with muscle weakness and diaphragmatic paralysis necessitating artificial ventilation and a tracheotomy (Bosch et al. 2011). Presenting symptoms of patients included in the review were severe axial and limb weakness, bulbar palsy, hearing loss, facial weakness and respiratory compromise (Bosch et al. 2012). Some patients with a molecularly confirmed diagnosis demonstrated abnormal plasma flavin levels and/or plasma acylcarnitine profiles, rapidly reversible with supplementation of riboflavin. Treatment with high dose oral riboflavin was effective and lifesaving. However, this review reported on patients with a possibly diverse etiology as only in 23/75 reported patients the diagnosis was confirmed with molecular analysis. Since then, more patients with a molecular diagnosis have been reported in the literature. Five years after the diagnosis of our index patient we performed a review of the literature to study the presentation, treatment and outcome of patients with a molecularly confirmed diagnosis of a riboflavin transporter deficiency aiming to extend our insight into this new inborn error of metabolism.

## Methods

A search was performed in Medline, Pubmed using the search terms ‘Brown-Vialetto-van Laere syndrome’ and ‘riboflavin transporter’. The publications found were studied for case reports of patients with a molecular diagnosis of a riboflavin transporter deficiency. All case reports were analyzed for clinical aspects, additional investigations, survival and treatment. Patients described more than one time in different reports were included once.

## Results

The search strategy identified 45 articles of which 19 articles reported on patients with a molecular diagnosis of a riboflavin transporter deficiency. After exclusion of duplicates 70 reported patients (42/70 (60 %) females and 28/70 (40 %)males) with either compound heterozygous or homozygous mutations in SLC52A2 or SLC52A3 were included in the review. Of these, 37 patients (53 %) were diagnosed with a RFVT2 deficiency (SLC52A2) (Johnson et al. 2012; Haack et al. 2012; Ciccolella et al. 2012; Foley et al. 2014; Srour et al. 2014; Menezes et al. 2016) and 33 (47 %) with a RFVT3 deficiency (SLC52A3) (Green et al. 2010; Johnson et al. 2010; Bosch et al. 2011; Anand et al. 2012; Koy et al. 2012; Dezfouli et al. 2012; Ciccolella et al. 2013; Bandettini Di Poggio et al. 2014; Spagnoli et al. 2014; Cosgrove et al. 2015; Davis et al. 2015; Horoz et al. 2015; Spagnoli and De Sousa 2012).

### Age and symptoms at presentation

The mean age of presentation was 4.1 years (range 0.25–27.0 years; SD 5.0) for the total group, 3.0 years (range (0.6–8.0 years; SD 1.7) for the RFVT2 deficient patients and 5.6 years (range 0.25–27.0 years; SD 7.0) for the RFVT3 deficient patients. Main presenting symptoms included hearing loss, weakness of limbs and neck, bulbar weakness leading to feeding difficulties, balance and gait problems and respiratory complaints like dyspnea or stridor. Less frequently visual problems and problems with speech were reported.

### Clinical features

During the course of illness most reported symptoms were cranial nerve deficits resulting in hearing loss and bulbar symptoms, weakness and sensory symptoms. Feeding difficulties and respiratory symptoms occurred secondary to bulbar and respiratory muscle weakness (Table 1). Remarkably, cognition was reported to be normal in 29 patients and no cognitive abnormalities were reported in any of the patients.

**Table 1:** Reported clinical features in patients with RFVT2 and RFVT3 deficiency

Clinical feature	RFVT2 deficiency (SLC52A2) *n* = 37	RFVT3 deficiency SLC52A3 *n* = 33	Total *n* = 70
Cranial nerve deficit	27 (73 %)	25 (76 %)	52 (74 %)
Hearing loss	33 (89 %)	25 (76 %)	58 (73 %)
Weakness	34 (92 %)	24 (73 %)	58 (73 %)
Sensory symptoms^a^	19 (51 %)	5 (15 %)	24 (34 %)
Feeding difficulties	9 (24 %)	8 (24 %)	17 (24 %)
Respiratory symptoms	21 (57 %)	25 (75 %)	46 (66 %)

^a^ Including ataxic gait without other cerebellar signs or muscle weakness in lower limbs in patients with confirmed sensorimotor axonal neuropathy

#### Cranial nerve deficits

Sensorineural hearing loss was reported in 58/70 patients at presentation or during the course of their disease. Abnormalities of all other cranial nerves were reported. It was not possible to specify the frequency of involvement of each cranial nerve, but bulbar symptoms due to dysfunction of cranial nerves VII-XII were reported in the majority of patients. Reduced vision and or optic atrophy were reported in 26/37 (70 %) patients with RFVT2 deficiency but only in 2/33 patients (6 %) with RFVT3 deficiency.

#### Weakness

Limb weakness (frequently accompanied by atrophy) was reported in 53/70 patients and described in most detail in patients with RFVT2 deficiency. In 34/37 (92 %) patients with RFVT2 deficiency the muscles in the upper extremities were more involved than the lower extremities and most patients suffered from shoulder girdle and distal hand muscle weakness. Many also developed axial weakness, with severe weakness of neck extensor and trunk muscles resulting in scoliosis in six patients (Johnson et al. 2012; Srour et al. 2014). In RFVT3 deficient patients more variable patterns of weakness were reported. Reflexes were reported in 25 RFVT2 deficient patients and were hypoactive or absent. Of four patients with RFVT3 deficiency two patients demonstrated normal reflexes, one hypoactive reflexes and one brisk deep tendon reflexes with an ankle clonus (Green et al. 2010).

#### Sensory symptoms

In a minority of cases sensory abnormalities were an isolated finding with a more severe disturbance of vibration- and position sense than loss of sensation for pain and touch (Srour et al. 2014). An ataxic gate was reported in 19/37 (51 %) patients with RFVT2 deficiency (Foley et al. 2014; Srour et al. 2014; Menezes et al. 2016). These patients had a confirmed sensorimotor axonal neuropathy and no weakness in the lower limbs or cerebellar signs, therefore a disturbed proprioception due to a progressive sensory neuropathy or neuronopathy seemed the cause of this gait disorder. In 5/33 patients with RFVT3 deficiency gait or truncal ataxia was described, without information on the presence or absence of a neuropathy (Green et al. 2010; Malafronte et al. 2013). Cerebellar signs were reported in one RFVT3 deficient patient described by Green et al.

#### Feeding difficulties

In 27 (RFVT2: 18; RFVT3: 9) patients information on feeding was provided (Johnson et al. 2010; Koy et al. 2012; Ciccolella et al. 2013; Foley et al. 2014; Bandettini Di Poggio et al. 2014; Spagnoli et al. 2014; Cosgrove et al. 2015). Feeding difficulties were present in 8/18 RFVT2 deficient patients and in 9/9 RFVT3 deficient patients. In 16/17 patients the feeding difficulties necessitated a gastronasal or PEG tube to secure feeding.

#### Respiratory symptoms

Respiratory problems were reported in 21/37 (57 %) patients with RFTV2 deficiency and in 25/33 (76 %) patients with RFVT3 deficiency (Green et al. 2010; Johnson et al. 2010; Bosch et al. 2011; Anand et al. 2012; Koy et al. 2012; Dezfouli et al. 2012; Johnson et al. 2012; Ciccolella et al. 2012; Ciccolella et al. 2013; Foley et al. 2014; Spagnoli et al. 2014; Cosgrove et al. 2015; Davis et al. 2015; Horoz et al. 2015). Respiratory symptoms developed during the course of the disease in most patients but were a presenting symptom in two and eight RFVT2 and RFVT3 deficient patients respectively. Diaphragm paralysis was documented in four patients with RFVT3 deficiency. Artificial respiratory support was required in 15/23 (65 %) RFVT2 deficient patients and in 9/22 (41 %) RFVT3 deficient patients.

### Biochemical abnormalities

Acylcarnitine profiles were reported in 28 RFTV2 deficient patients and in 8 RFVT3 patients and were abnormal in 17/28 (61 %) and 4/8 (50 %) respectively. All abnormal acylcarnitine profiles normalized rapidly with riboflavin supplementation.

### Additional investigations

#### MRI

In 16 (RFVT2:6; RFVT3:10) patients a cranial MRI was made. All cranial MRI’s in RFVT2 deficient patients were normal. Abnormalities were found in 2/10 patients with RFVT3 deficiency: one patient demonstrated increased signal intensities of the vestibular nuclei, the cerebellar superior peduncle and central tegmental tract (Koy et al. 2012), another showed symmetrical, hyperintense areas in the middle cerebellar peduncles on T2 FLAIR images (Bandettini Di Poggio et al. 2014).

#### Neurophysiology

An EMG was performed in 42 patients (SLC52A2: 32; SLC52A3: 10). In 32/32 (100 %) RFVT2 deficient patients and 2/10 RFVT3 deficient patients the authors reported findings consistent with a sensorimotor axonal neuropathy. In the majority of RFVT2 deficient patients sensory findings were more pronounced than motor findings. Sensory nerve action potentials (SNAP) were absent in most patients and compound motor action potential (CMAP) amplitudes were absent or decreased in arms and legs. Reported motor nerve conduction velocities were mildly slowed or normal. In 1/10 RFVT3 deficient patients the neurophysiological exam was normal, in 7/10 described as motor neuropathy or motor neuron disease.

#### Histopathology

A sural nerve biopsy, taken in 8 RFVT2 deficient patients showed signs of a chronic axonal neuropathy in 7/8 patients with preferential loss of large diameter myelinated axons, in accordance with the clinical finding of sensory ataxia (Johnson et al. 2012; Haack et al. 2012; Foley et al. 2014; Srour et al. 2014). A sural nerve biopsy taken from a patient with RFVT3 deficiency revealed signs of a motor neuronopathy and axonal degeneration (Johnson et al. 2010).

## Treatment

A total of 31 patients was not treated with riboflavin supplementation and these patients all demonstrated a gradual deterioration (Green et al. 2010; Johnson et al. 2010; Dezfouli et al. 2012; Johnson et al. 2012; Ciccolella et al. 2012; Malafronte et al. 2013; Ciccolella et al. 2013; Bandettini Di Poggio et al. 2014; Srour et al. 2014). Fifteen patients (RFVT2: 6; RFVT3: 9) were reported to have died at the time of publication. The cause of death was reported in nine patients, seven patients died of respiratory insufficiency, and the cause of death was not clearly reported in 2. Mean age between onset of symptoms and death was 7.11 years (range 0.6–19.0 years).

In 39 (RFVT2: 27; RFVT3: 13) patients treatment with oral riboflavin was started in a dosage ranging from 7 to 60 mg/kg/day (Bosch et al. 2011; Anand et al. 2012; Koy et al. 2012; Haack et al. 2012; Foley et al. 2014; Spagnoli et al. 2014; Cosgrove et al. 2015; Menezes et al. 2016; Davis et al. 2015; Horoz et al. 2015). Side effects were reported in three patients and included gastrointestinal symptoms. No death after the start of treatment has been reported. Clinical response was reported in 20 RFVT2 deficient patients. Twelve patients (60 %) improved, eight patients (40 %) remained stable after initiation of riboflavin. In one RFVT3 deficient patient riboflavin was stopped after 1 week because of lack of a clinical response. In ten patients with RFVT3 deficiency clinical response was documented. Eight improved (80 %), 2 (20 %) remained stable. Improvement occurred within days to months in both types of riboflavin deficiency. In the remaining patients the effect of riboflavin was not mentioned.

In 15 RFVT2 deficient patients age at the start of treatment with riboflavin and response to treatment were reported (Foley et al. 2014). Mean time between onset of symptoms and start of treatment in the RFVT2 deficient patients that remained stable after initiation of riboflavin was 19.2 years (range 1.9–50.0 years, SD 16.9), in the patients that improved 7.4 years (range 0.5–20.6 years, SD 7.3). Information on time between onset of symptoms and start of treatment and response to treatment was available in 6 RFVT3 deficient patients (Bosch et al. 2011; Anand et al. 2012; Ciccolella et al. 2013; Spagnoli et al. 2014). Mean time between onset of symptoms in the five patients that improved was 3.1 years (range 0.1–14 years, SD 6.1). One patient did not improve after 1 year of treatment with riboflavin (Davis et al. 2015).

## Discussion

We performed a review of the literature aiming to obtain better insight in the presentation, treatment and outcome of patients with a molecularly confirmed diagnosis of a riboflavin transporter deficiency type two (RFVT2) or three (RFVT3). Including the first reports of riboflavin transporter deficiencies in 2010, 70 case reports have been published.

The presenting age of the patients was variable with the oldest patient presenting at the age of 27 years but most patients presenting in the first years of life. Reported clinical symptoms were hearing loss, weakness, cranial nerve deficits, sensory symptoms including sensory ataxia, feeding difficulties and respiratory problems (frequently due to diaphragmatic paralysis), which was comparable to our previous review of a possibly more heterogeneous cohort of patients (Bosch et al. 2012). No cognitive abnormalities were reported in any of the patients. Our results confirm the proposal of Foley et al. (2014) that sensory ataxia and optic atrophy seem more prevalent in RFVT2 than RFVT3 deficient patients (62 % vs 6 % and 70 % vs 6 % respectively). However, patient numbers are small and not all symptoms may have been reported for all patients. Future studies are necessary to further explore differences between RFVT2 and RFVT3 deficiency.

While starkly abnormal acylcarnitine profiles led to the diagnosis in our index patient, only approximately half of the reported patients demonstrated abnormalities in their acylcarnitine profile at diagnosis which confirms our previous report that acylcarnitine profiles are not a valid instrument for the diagnosis of riboflavin transporter deficiencies (Bosch et al. 2011; Bosch et al. 2012). In the past 5 years more insight has been obtained into the pathophysiology of this disorder. Neurophysiological and histopathological studies almost invariably demonstrated findings consistent with an axonal sensorimotor peripheral neuropathy in RFVT2, confirming the report of Foley et al. (2014). Data on neurophysiology and histopathology in RFVT3 are scarce but point to a sensorimotor neuropathy in some patients. In addition clinical findings in both RFVT2 and RFVT3 indicate involvement of cranial nerves. The underlying pathophysiological mechanism of this neuropathy or neuronopathy remains to be elucidated. Only one RFVT3 deficient patient showed signs of upper motor neuron involvement.

Treatment with oral riboflavin supplementation (doses varying from 7 to 60 mg/kg/day) was initiated in 39/70 patients. All 31/70 untreated patients suffered from progression of the disease, resulting in death (mostly from respiratory insufficiency) in 15/31 patients at the time of the publication. In contrast, no death was reported in the cohort of 39 treated patients and in those patients in whom effects of treatment were reported 71 % demonstrated clinical improvement and 29 % a stabilization of symptoms. Clinical improvement may occur within days to months after start of treatment and an earlier start of treatment seems associated with better and more rapid improvement suggesting that prolonged disease may result in irreversible damage.

### Challenges

At this time our insight into these severe neurodegenerative disorders is very limited and many questions remain to be answered. The exact pathophysiology is still unclear. The spectrum of disease is unknown and while most reported patients were diagnosed with severe disease at a very young age, there may well exist a larger cohort of patients with a milder phenotype who might benefit greatly from early treatment. The optimal dosing of oral riboflavin is unknown and patients have been reported to recover well on doses varying from 7 to 60 mg/kg/day. In the 16 patients that received riboflavin therapy described in the study by Foley et al. doses varied between 10 and 60 mg/kg/day. In the majority of these patients riboflavin was started at a dose of 20–40 mg/kg/day. Presently, the authors prescribe 20 mg/kg/day with a maximum of 3dd 400 mg. Ultimately, it is not clear whether the riboflavin supplementation will be effective in preventing symptoms for life or whether it may simply delay the occurrence of symptoms, especially in our youngest patients.

Until now the disease course of our index patient is encouraging. After presenting at the age of 6 months with severe generalized weakness and diaphragmatic paralysis necessitating artificial ventilation our patient slowly recovered after the start of riboflavin supplementation (Bosch et al. 2011). After 16 months of treatment with oral riboflavin in a dose of 10 mg/kg/day (age 22 months) he was able to walk independently. Respiratory support could be decreased over time and the tracheotomy was closed at age 4 years. At age nine he demonstrates a fully normal neurological examination and normal strength. His sister, presenting at age 3 months with axial weakness, recovered within 7 days of start of treatment and demonstrates a normal development at age 6 years.

## Conclusion

The riboflavin transporter deficiencies present with cranial nerve deficits including hearing loss, weakness, sensory symptoms including sensory ataxia, feeding difficulties and respiratory difficulties which are caused by a progressive axonal sensory and motor peripheral neuropathy and a cranial neuropathy. The diagnosis can only be made through molecular analysis of all genes. Treatment with oral supplementation of riboflavin is lifesaving. Therefore, treatment must be started immediately if a riboflavin transporter deficiency is suspected, without first awaiting the results of molecular diagnostics.
